# Effect of music therapy on patient experience in gastrointestinal endoscopy: a scoping review

**DOI:** 10.1093/jcag/gwaf034

**Published:** 2025-12-22

**Authors:** Jason Hearn, Stephanie Carpentier

**Affiliations:** Department of Internal Medicine, Dalhousie University, Saint John, NB, E2L 4L5, Canada; Department of Medicine, Saint John Regional Hospital, Saint John, NB, E2L 4L2, Canada; Department of Internal Medicine, Dalhousie University, Saint John, NB, E2L 4L5, Canada; Department of Medicine, Saint John Regional Hospital, Saint John, NB, E2L 4L2, Canada; Division of Gastroenterology, Saint John Regional Hospital, Saint John, NB, E2L 4L2, Canada

**Keywords:** gastroenterology, music therapy, patient-reported outcomes, pain, anxiety, endoscopy

## Abstract

**Background:**

Music therapy is a low-cost and low-risk intervention that has been shown to improve patient-reported outcome measures (PROMs) and patient-reported experience measures (PREMs) in various areas of medicine including gastrointestinal (GI) endoscopy. A scoping review was performed to answer the following research question: *What is known from the existing literature about the effect of music therapy used in adult GI endoscopy on PROMs (eg pain, anxiety) and PREMs (eg satisfaction, willingness to repeat procedure)?*

**Methods:**

Guided by the methodologic framework proposed by Arksey and O’Malley, 3 medical databases were queried for articles pertinent to the research question and published between January 2005 and December 2024. Studies were selected for inclusion based on established criteria and summarized in a comprehensive data table as well as accompanying figures.

**Results:**

A total of 30 original research articles were selected for inclusion. The most reported outcomes were pain (*N* = 21), anxiety (*N* = 21), and satisfaction (*N* = 14). Significant improvements following music therapy were described most commonly for anxiety (*N* = 15, 71% of 21) and satisfaction (*N* = 10, 71% of 14) and less commonly for pain (*N* = 11, 52% of 21). Reductions in pain and anxiety were more consistent for music interventions performed in the pre-endoscopy period.

**Conclusions:**

Music therapy appears to be an effective means of improving anxiety and satisfaction in patients undergoing GI endoscopy. Endoscopists should consider music therapy as a non-pharmacologic adjunct to improve the patient experience in endoscopy.

## Introduction

Patient-reported outcome measures (PROMs) and patient-reported experience measures (PREMs) are simple and effective means of quantifying the patient experience and identifying ways in which we can provide more patient-centred care.^1^ PROMs include instruments completed by the patient that measure their health status (eg pain, anxiety), whereas PREMs are tools that capture how a patient experiences healthcare (eg satisfaction, willingness to repeat a procedure).^2^ The importance of such metrics has been increasingly recognized over time, as evidenced by the rising number of clinical trials targeting patient-reported measures as endpoints.^3^

Music therapy is a non-pharmacologic tool that has been shown to improve patient-reported measures in a wide breadth of clinical settings, including operating rooms,^4^ intensive care units,^5^ and palliative care environments.^6^ Gastrointestinal (GI) endoscopy presents an area in which music therapy could make a significant impact, particularly given the large number of these procedures performed each year^7^^,^^8^ and the pre-procedural anxiety often faced by patients.^9^^,^^10^ Previous review articles have investigated the effect of music therapy on a variety of outcomes in esophagogastroduodenoscopy (EGD), colonoscopy, and sigmoidoscopy and have demonstrated variable results in terms of effectiveness.^11–13^ However, these previous studies have generally focused their reviews on one type of GI endoscopy and have broadly examined the effectiveness of music therapy without a specific emphasis on patient-reported measures. As such, we performed a scoping review to summarize the effect of music therapy on patient-reported measures in patients undergoing any type of GI endoscopy.

## Methods

### Study design

Guided by the methodological framework for scoping reviews proposed by Arksey and O’Malley^14^ and the Preferred Reporting Items for Systematic reviews and Meta-Analysis extension for Scoping Reviews (PRISMA-ScR),^15^ a scoping review was performed to study the following research question: *What is known from the existing literature about the effect of music therapy used in adult GI endoscopy on PROMs (eg pain, anxiety) and PREMs (eg satisfaction, willingness to repeat procedure)?* The completed PRISMA-ScR checklist summarizing the comprehensive reporting of our findings is included in Appendix S1.

### Search strategy

Based on a preliminary review of the existing literature, a search strategy was developed to capture the 3 main aspects of the research question: *music therapy*, *GI endoscopy*, and *patient-reported measures*. Keywords identified for each of the 3 components are summarized in Table 1. Using the devised search strategy, a search was performed on 3 pertinent databases (PubMed, Cumulative Index to Nursing and Allied Health Literature [CINAHL], and Embase) for articles that contained at least one search term from each column in Table 1 and were published in a 20-year period between January 1, 2005, and December 31, 2024. The exact search strategy is included in Appendix S2, with the date constraints being applied using the functionality on each individual database.

**Table 1.** Search terms used for each aspect of the research question.

Music therapy	GI endoscopy	Patient-reported measures
music	gastroenterology, gastrointestinal, endoscop*, gastroscop*, EGD, gastrofibroscop*, esophagogastroduodenoscop*, cholangiopancreatograph*, ERCP, enteroscop*, duodenoscop*, esophagoscop*, colonoscop*, sigmoidoscop*, proctoscop*, polypectomy	pain, analgesia, satisfaction, dissatisfaction, anxiety, stress, comfort, discomfort, emotion, tolerance, perception

### Inclusion and exclusion criteria

Studies were included in the scoping review if they were original research articles available in English that examined the direct effect of music therapy on patient-reported measures in GI endoscopy. We excluded studies in pediatric populations, studies that lacked a control group, literature reviews, protocols, posters, abstracts, and research theses.

### Study selection

All studies identified using the search strategy were imported into Covidence (Veritas Health Innovation, Australia) for review, with duplicate articles automatically being removed. As a first pass, we assessed titles and abstracts for relevance based on the inclusion and exclusion criteria, and removed studies deemed to be irrelevant. In the second phase, we reviewed full-text manuscripts for appropriateness based on the established criteria. Articles meeting the criteria based on full-text review were included in data extraction, whereas articles failing to meet these criteria were tagged with a reason for exclusion.

### Data extraction

A standardized data table was developed to extract information from the selected studies. This table included the following fields: primary author, publication year, GI procedure, country, description of the study groups, type of music, mode of delivery of music, outcome measures, and key findings. Study group sizes were indicated as the number of patients that were included in the final data analysis. Intervention groups that were not exposed to a music therapy intervention were not included in the table. Outcome measures and associated findings that were not patient-reported measures (eg procedure difficulty, sedation use, vital signs) were not included in the table. For each article, a binary number was assigned to each patient-reported measure described in the manuscript indicating whether a significant result was demonstrated relative to a control group.

## Results

**Figure 1.**
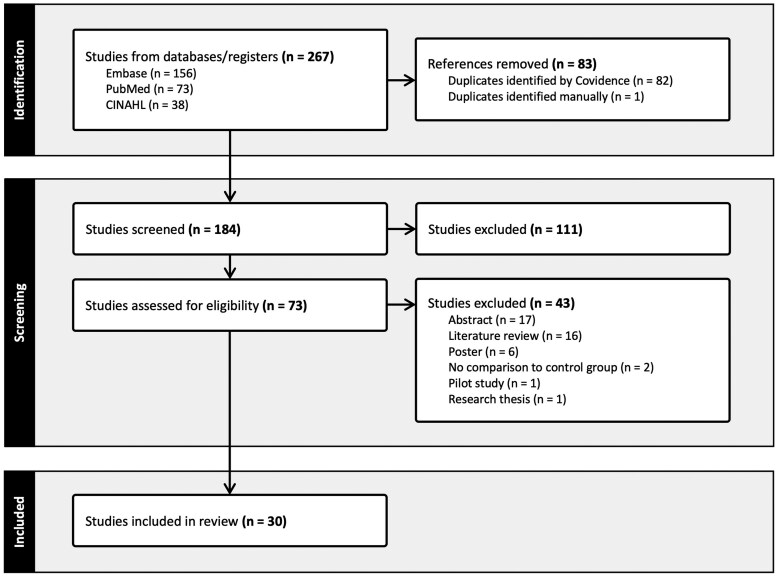
PRISMA-ScR flowsheet summarizing study selection process.

Searches of the PubMed, CINAHL, and Embase databases returned a total of 267 articles (73, 38 and 156 articles, respectively), which was reduced to an extracted sample of 184 after removal of all duplicates. Initial screening of these studies based on their title and abstract identified 73 articles that met the inclusion and exclusion criteria. In evaluating the full text of the remaining articles, a total of 30 studies were ultimately selected for inclusion in the scoping review. Articles were excluded at this stage for a variety of reasons, including lack of a control group (*N* = 2) or studies in the form of an abstract (*N* = 17), literature review (*N* = 16), poster (*N* = 6), pilot study (*N* = 1), or research thesis (*N* = 1). The PRISMA-ScR flowsheet summarizing the selection process is presented in Figure 1. Appendix S3 presents a data table summarizing the selected articles.

**Figure 2.**
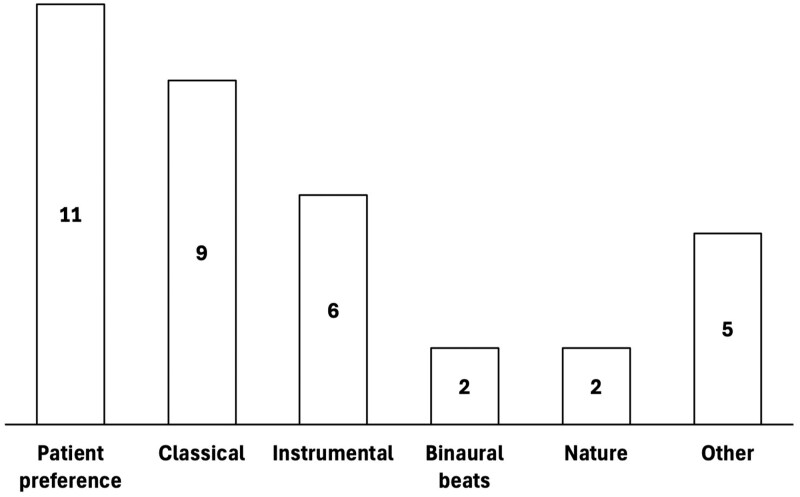
Music styles included in the selected studies.

The bulk of the included studies were performed in Europe (*N* = 15) and Asia (*N* = 13), with the other 2 studies occurring in the United States (*N* = 1) and Australia (*N* = 1). Studies assessed the effect of music therapy in both lower endoscopy (*N* = 24) and upper endoscopy (*N* = 9). A variety of music types were used in the selected studies (as summarized in Figure 2), most notably patient-selected music (*N* = 11), classical music (*N* = 9), instrumental music (*N* = 6), binaural beats (*N* = 2), and nature sounds (*N* = 2). The main modes of music delivery were headphones (*N* = 23) and speaker (*N* = 5).

**Figure 3.**
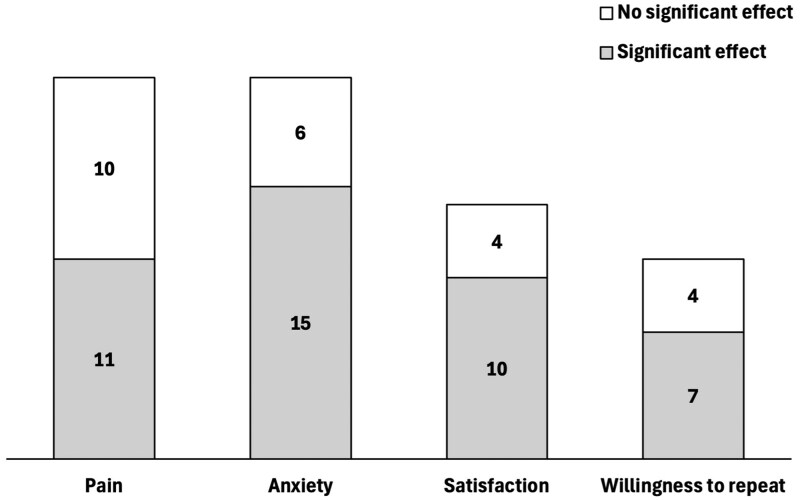
Patient-reported measures included in the selected studies, where the filled portion of the bar indicates the number of studies in which music therapy was associated with a significant improvement in the patient-reported measure.

A variety of patient-reported measures were assessed in the selected studies, most notably pain (*N* = 21), anxiety (*N* = 21), satisfaction (*N* = 14), and willingness to repeat the procedure (*N* = 11). As depicted in Figure 3, the outcomes most commonly associated with a significant improvement following a music therapy intervention were anxiety (*N* = 15, 71% of 21), satisfaction (*N* = 10, 71% of 14), and willingness to repeat the procedure (*N* = 7, 64% of 11). Just over half of the studies assessing the effect on pain reported a significant reduction following music therapy (*N* = 11, 52% of 21).

### Music therapy in the pre-endoscopy period

Several studies investigated the use of music therapy prior to endoscopy, many of which focused on the associated effect on anxiety. Aksu had patients listen to Turkish classical music for 45 minutes prior to EGD and found that both pain and anxiety were significantly reduced relative to a control group.^16^ A second study in Turkey showed the administration of binaural beats for 15 minutes before EGD to be associated with significant improvements in anxiety and satisfaction.^17^ Two studies assessed the effect of listening to patient-selected music for 15 minutes prior to endoscopy and identified a significant reduction in anxiety when compared to controls.^18^^,^^19^ A Japanese group exposed patients to an “optimal soothing environment” comprising comforting images, sounds, and smells prior to EGD, which led to improvements in anxiety when compared to a control group.^20^ Padam et al. investigated the effect of 2 distinct music therapy interventions prior to EGD (Vedic chants and Indian classical music) and showed both groups had reduced anxiety levels when compared to a control group.^21^ The results of a second Indian study were less clear. Sobana et al. described a significant reduction in anxiety following a period of listening to music for 10 minutes prior to EGD; however, tables within the manuscript demonstrate a contradictory increase in anxiety scores over this period.^22^ Given the lack of clarity, this article (and the claimed significance of the results) was omitted in the formulation of Figure 3. A final Chinese study showed two music interventions (classical music and pop music) for 20 minutes prior to colonoscopy to be associated with increased patient satisfaction when compared to a control group.^23^

### Music therapy in the endoscopy period

A total of 12 articles examined the effect of music during endoscopy, with a greater number of studies focusing on pain in addition to anxiety. All studies in this group investigated the effects during lower endoscopy, while 2 articles included patients undergoing upper and/or lower endoscopy. Two studies that played patient-selected music during colonoscopy identified significant improvements in pain and willingness to repeat the procedure when compared to control groups.^24^^,^^25^ Two studies investigated the effects of music therapy in patients undergoing upper and/or lower endoscopy receiving different types of sedation (conscious sedation and deep sedation) and found significant reductions in pain and anxiety when comparing intervention and control groups that received either sedation type.^26^^,^^27^ Çelebi et al. demonstrated a significant improvement in pain and anxiety through exposure to Turkish classical music during colonoscopy.^28^ Similar improvements in pain, anxiety, satisfaction, and willingness to repeat the procedure were exhibited by Sun et al. in patients listening to light piano music during colonoscopy.^29^

In contrast to the aforementioned studies, several articles failed to demonstrate a significant effect of music therapy during endoscopy on pain. Björkman et al. administered instrumental music during colonoscopy and found no significant between-group difference in terms of pain, though the authors did report a significant reduction in anxiety among women in the intervention group.^30^ Kartin et al. combined pre-endoscopy meditation with patient-selected music during colonoscopy and identified a significant reduction in anxiety within the intervention group but no improvement in terms of pain.^31^ Two further studies showed no significant pain reduction in patients undergoing colonoscopy while listening to music of their choosing.^32^^,^^33^

Two additional studies investigated the effects of music therapy on non-pain outcomes in colonoscopy. Walter et al. studied the effect of patient-selected music on satisfaction in German patients undergoing colonoscopy and demonstrated no significant between-group difference.^34^ Ko et al. identified a reduction in anxiety amongst patients listening to either informal classical music or light music during colonoscopy; however, the reduction was not statistically significant.^35^

### Music therapy in both the pre-endoscopy and endoscopy periods

Ten studies investigated the effect of music therapy used both prior to and during colonoscopy, with fewer studies demonstrating significant results. Li et al. assessed 2 different audio interventions (relaxing music and recordings of yoga nidra) for 10 minutes before and during colonoscopy, and both interventions were associated with significant improvements in pain, satisfaction, and willingness to repeat the procedure relative to a control group.^36^ Two Turkish groups had patients listen to classical music before and during colonoscopy and demonstrated significant improvements in pain and satisfaction when compared to control groups.^37^^,^^38^ Tani et al. exposed patients to binaural beats for 5 minutes before and during colonoscopy and identified significant improvements in pain, satisfaction, and willingness to repeat the procedure.^39^

Several studies experimenting with music therapy in the pre-colonoscopy and colonoscopy periods failed to demonstrate significant improvements in a variety of patient-reported measures. Ko et al. administered a series of 15 “easy listening” songs for 20 minutes before and during colonoscopy and demonstrated significantly higher satisfaction levels but no significant improvements in pain or anxiety.^40^ An Australian group played music by Johann Sebastian Bach for 10 minutes prior to and during colonoscopy and failed to identify any significant change in terms of pain, anxiety, or satisfaction.^41^ Four studies assessed the effect of various forms of relaxing music before and during colonoscopy, including music by Enya,^42^ instrumental music with integrated nature sounds,^43^^,^^44^ and “calming soft melodies.”^45^ None of these studies showed significant between-group difference in terms of pain^42–45^; however, 2 of the articles reported a significant improvement in anxiety.^44^^,^^45^

## Discussion

This scoping review highlights existing research into the effect of music therapy on patient-reported measures in GI endoscopy. Music therapy interventions have been shown to improve the patient experience in both upper and lower endoscopy, as well as in various geographic locations. Several patient-reported measures have been studied in this setting, most notably pain, anxiety, satisfaction, and willingness to repeat the procedure.

In general, pain was the patient-reported measure that was least frequently reported to improve following music therapy. A slightly higher proportion of music interventions in the pre-endoscopy setting were associated with a significant change in pain scores. As well, all 3 studies that studied the effect on pain in upper endoscopy reported significant improvements. Despite these subgroups in which the effect was slightly more consistent, pain appears to be the patient-reported measure that is least likely to be affected by music therapy. This conclusion is supported by 2 previous meta-analyses that identified non-significant effects of music therapy on pain in patients specifically undergoing sigmoidoscopy^11^ and colonoscopy.^13^

Anxiety was the outcome most commonly associated with a significant improvement following a music intervention. This effect was particularly notable in the pre-endoscopy setting, as all 6 studies that assessed the effect of a music intervention prior to endoscopy on anxiety reported a significant change. Similarly, all 8 studies assessing the effect on anxiety in upper endoscopy reported significant improvements. In contrast, the frequency of a significant effect on anxiety was lower for interventions using music both prior to and during endoscopy, as well as in studies involving lower endoscopy. This general effectiveness of music therapy on anxiety has been reported previously for different forms of GI endoscopy.^11^^,^^12^^,^^46^ The apparent increased effect of music used prior to endoscopy may be explained by the large proportion of patients that experience pre-endoscopy anxiety^9^^,^^10^ and thus may stand to benefit from interventions targeting anxiety at this time point.

Patient satisfaction was also regularly reported to be improved by music therapy in the study. This effect was relatively consistent regardless of the timing of the music therapy intervention, though a slightly more reliable effect was demonstrated for patient undergoing lower endoscopy. Similarly, patient willingness to repeat the procedure was increased in the majority of studies, with the small number of studies making it difficult to draw conclusions regarding subgroups. This general acceptance of music therapy and resulting effect on satisfaction outcomes correlate with previous literature regarding patients undergoing various forms of GI and non-GI endoscopy.^46^

The potential clinical implications of our research findings are numerous. Endoscopic procedures can be uncomfortable experiences for patients, whether it be through physical or emotional discomfort.^47^ Music therapy may stand to mitigate patient discomfort as it relates to endoscopy, given the possible effects on anxiety, satisfaction, and—to a lesser extent—pain. Anxiety and stress regarding the procedure have also been identified as barriers for patients considering colonoscopy surveillance.^48^ Thus, the potential improvements in patient anxiety and willingness to repeat endoscopy could increase adherence to endoscopic surveillance protocols. These potential benefits are particularly attractive given the minimal added risk associated with music therapy.

Despite the promising results of the presented studies, several shortcomings were identified in the existing literature. First, the size of the intervention groups in the selected studies were generally small, with a mean size of 74 participants and maximum size of 169 participants. Moreover, none of the studies were performed in Canada and only one study was performed in North America, limiting the generalizability of the results to the Canadian context. A large majority of studies examined music delivered via headphones, a decision that was perhaps made to allow for blinding of the endoscopy team. However, headphones are less practical for widespread use in the endoscopy suite given the frequent need for communication between the endoscopy team and the patient, potentially affecting the applicability of the results. Finally, the identified studies are heterogenous in multiple domains, included the implemented music style, mode of delivery, time at which music was used, and time at which outcomes were measured. The complexity and diversity of the current literature, as well as the limited number of identified studies, would make a high-quality systematic review impractical at this time.

The conclusions of this study must be considered alongside its limitations. Given the implemented framework, the selected studies were not assessed in terms of methodological quality. Our search was limited to articles published in English; thus, we potentially omitted important results published in other languages. Due to resource constraints, we also limited our search to 3 representative databases. Although we selected these databases based on their broad and comprehensive indexing of medical research articles, this narrowing of our search strategy could have excluded relevant studies. We also did not hand-search reference lists from excluded review articles, which could have increased the number of articles included in the review. Lastly, articles were reviewed by a single member of the research team (J.H.), which may have introduced unintentional bias.

## Conclusions

Music therapy is a simple, low-cost and low-risk intervention with the potential to improve the experience of patients undergoing GI endoscopy. A scoping review was performed and identified 30 studies investigating the effect of music therapy on patient-reported measures in patients undergoing GI endoscopy. The results of the selected studies suggest a general effectiveness of music therapy in improving patient anxiety, satisfaction, and willingness to repeat the procedure. A reduction in pain has also been reported in several studies; however, this effect appears to be less reliable. Anxiety and pain were more consistently improved by music interventions performed in the pre-endoscopy period. Given the potential benefits and minimal associated risks, endoscopists should consider music therapy as a non-pharmacologic adjunct to improve patient experience in endoscopy.

## Supplementary Material

gwaf034_Supplementary_Data

## Data Availability

All relevant data are within the manuscript and appendices.
