# Supplementary material for: Effect of music therapy on patient experience in gastrointestinal endoscopy: a scoping review
Source: J Can Assoc Gastroenterol. 2025 Dec 22;9(1):4–10. doi: 10.1093/jcag/gwaf034 (PMC12884849; doi:10.1093/jcag/gwaf034)
Supplement: gwaf034_Supplementary_Data [file gwaf034_supplementary_data.zip › gwaf034_Supplementary_Data/S2 Appendix.docx]

**S2 Appendix. Exact text inputted into database search engines.**

(music) AND (endoscop* OR gastroscop* OR EGD OR gastrofibroscop* OR esophagogastroduodenoscop* OR cholangiopancreatograph* OR ERCP OR enteroscop* OR duodenoscop* OR esophagoscop* OR colonoscop* OR sigmoidoscop* OR proctoscop* OR polypectomy) AND (pain OR analgesia OR satisfaction OR dissatisfaction OR anxiety OR stress OR comfort OR discomfort OR emotion OR tolerance OR perception)
