# Supplementary material for: Effect of music therapy on patient experience in gastrointestinal endoscopy: a scoping review
Source: J Can Assoc Gastroenterol. 2025 Dec 22;9(1):4–10. doi: 10.1093/jcag/gwaf034 (PMC12884849; doi:10.1093/jcag/gwaf034)
Supplement: gwaf034_Supplementary_Data [file gwaf034_supplementary_data.zip › gwaf034_Supplementary_Data/S3 Appendix.docx]

**S3 Appendix.** Study characteristics for music therapy interventions in GI endoscopy, where CG = control group; EGD = esophagogastroduodenoscopy; IG = intervention group; LS = Likert scale; NRS = numeric rating scale; STAI = Spielberger State-Trait Anxiety Inventory; and VAS = visual analog scale.

| **Author, *Year*** | **Procedure, *Country*** | **Study Groups** | **Type of Music, *Mode of Delivery*** | **Outcome, *Measurement*** | **Key Findings** |
| --- | --- | --- | --- | --- | --- |
| Aksu, *2023* | EGD, *Turkey* | CG (N = 40) - Underwent routine EGD. IG (N = 40) - In addition to CG care, listened to music for 45 minutes before procedure. | Büzürk mode (makam) of classical Turkish music, *Headphones.* | Anxiety, *STAI* Pain, *VAS* Comfort, *VAS* | Pain and anxiety were significantly reduced in IG when compared to CG. Comfort was increased significantly in IG when compared to CG. |
| Bashiri, *2018* | EGD and/or colonoscopy, *Turkey* | All groups underwent routine EGD and/or colonoscopy, with differences outlined below. CG-A (N = 25) - Conscious sedation without music. CG-B (N = 55) - Deep sedation without music. IG-A (N = 33) - Conscious sedation with music. IG-B (N = 41) - Deep sedation with music. | Patient's favourite genre of music, *Headphones.* | Anxiety, *STAI* Pain, *NRS* Satisfaction, *LS* | Pain increased significantly from pre- to post-procedure in CG-A and CG-B, whereas pain was not significantly different from pre- to post-procedure in IG-A and IG-B. Anxiety was significantly reduced from pre- to post-procedure in CG-B, IG-A and IG-B, though the effect was more significant in IG-A and IG-B. No significant between-group difference was identified in satisfaction. No sex-based differences were identified. |
| Bechtold, *2006* | Colonoscopy, *United States* | CG (N = 81) - Underwent routine colonoscopy. IG (N = 85) - In addition to CG care, listened to music before and during procedure. | Relaxing music (Watermark by Enya), *Speaker.* | Pain, *VAS* Satisfaction, *Two LS and one VAS* | Patients reported better overall satisfaction on all three scales in IG when compared to CG, with two of the three differences being significant. No significant between-group difference was identified in pain. |
| Bjorkman, *2013* | Colonoscopy, *Sweden* | CG (N = 60) - Underwent routine colonoscopy. IG (N = 60) - In addition to CG care, listened to music during the procedure. | Instrumental music with tempo of 60-80 beats per minute, *Headphones.* | Anxiety, *STAI and VAS* Pain, *VAS* Relaxation, *VAS* Well-being, *VAS* | Anxiety was significantly reduced amongst women in IG when compared to women in CG. Well-being was significantly increased in IG when compared to CG, with a larger effect seen in men. Relaxation was improved amongst men in IG when compared to CG, but the difference was not significant. No significant between-group difference was identified in pain. |
| Brix, *2022* | Colonoscopy, *Denmark* | CG (N = 168) - Underwent routine colonoscopy. IG (N = 169) - In addition to CG care, listened to music in the waiting room and during the procedure. | Instrumental acoustic music with integrated sounds of nature, *Headphones (waiting room) and pillow with built-in speaker (procedure).* | Anxiety, VAS Pain, *NRS* Worst recalled pain intensity, *NRS* Satisfaction, *LS* | No significant between-group differences were identified in pain, anxiety, worst recalled pain intensity or satisfaction. |
| Cakir, *2023* | Colonoscopy, *Turkey* | CG (N = 30) - Underwent routine colonoscopy. IG (N = 30) - In addition to CG care, listened to music before and during the procedure. | Acemasiran-type classical Turkish music, *Headphones.* | Anxiety, *STAI* Pain, *VAS* Satisfaction, *VAS* | Pain was significantly reduced both during and following the procedure in IG when compared to CG. No significant between-group difference was identified in anxiety. Satisfaction was significantly higher in IG when compared to CG. Effectiveness of distraction using a stress ball and virtual reality application was also assessed, but not felt to be relevant to this review. |
| Çelebi, *2020* | Colonoscopy, *Turkey* | CG (N = 56) - Underwent routine colonoscopy. IG (N = 56) - In addition to CG care, received 30 minutes of music therapy during colonoscopy. | Ajam Ashiran maqam of classical Turkish music, *Headphones.* | Anxiety, *STAI* Pain, *VAS* Comfort, VAS | Pain and anxiety were significantly lower in IG when compared to CG. Comfort was significantly higher in IG when compared to CG. Comparison of scores from pre- to post-colonoscopy revealed that patients in IG had reductions in both pain and anxiety following the procedure; whereas patients in CG had increases in both of these metrics following the procedure. |
| Costa, *2010* | Colonoscopy, *Italy* | CG (N = 53) - Underwent routine colonoscopy while wearing "mute" headphones to ensure blindness of physicians and nurses.  IG (N = 56) - Underwent routine colonoscopy while listening to music therapy during the procedure. | Choice of wide range of genres (e.g. blues, classic, country, jazz, reggae, pop, etc.), *Headphones* | Pain, *VAS* Satisfaction, *Qualitative rating* Willingness to repeat procedure, *Qualitative rating* | Pain was significantly reduced in IG when compared to CG. This trend was seen in both males and females, though reduction amongst females was less pronounced and failed to reach statistical significance. Satisfaction and willingness to repeat the procedure were significantly greater in IG when compared to CG. |
| De Silva, *2016* | Colonoscopy, *Sri Lanka* | CG (N = 67) - Underwent routine colonoscopy. IG (N = 67) - In addition to CG care, listened to music during colonoscopy. | Choice of songs from a variety of genres (e.g. Sinhala, Hindi, classical, hip hop), *Headphones* | Pain, *VAS* Willingness to repeat procedure, *LS* | Pain was significantly reduced in IG when compared to CG. Patients were significantly more likely to express a willingness to undergo a repeat procedure in IG when compared to CG. Effectiveness of visual distraction by watching a movie during colonoscopy was also assessed, but not felt to be relevant to this review. |
| Demirci, *2024* | EGD, *Turkey* | CG (N = 48) - Underwent routine EGD without sedation. IG (N = 48) - In addition to CG care, listened to music for 15 minutes prior to the procedure. | Binaural beats with relaxing theta waves at 5 Hertz, *Headphones* | Anxiety, *STAI* Satisfaction, *Quantitative rating* Willingness to repeat procedure, *Qualitative rating* | Anxiety decreased significantly from pre- to post-procedure in IG, whereas no significant change was identified in CG. When compared to CG, a significantly higher proportion of patients in IG indicated that they would repeat an endoscopic procedure without sedation. No significant between-group differences were identified in patient satisfaction. |
| El-Hassan, *2009* | EGD and/or lower endoscopy, *United Kingdom* | CG (N = 88) - Underwent routine EGD and/or lower endoscopy. IG (N = 92) - In addition to CG care, listened to self-selected music therapy for 15 minutes prior to endoscopy. | Choice of wide range of genres (e.g. classical, jazz, rock, country, etc.), *Headphones* | Anxiety, *STAI* | The reduction in anxiety from pre- to post-intervention was significantly greater in IG when compared to CG. This decrease in anxiety remained significant even when controlling for age (less than or greater than 51 years) and procedure type. |
| Harikumar, *2006* | Colonoscopy, *India* | CG (N = 40) - Underwent routine colonoscopy while wearing headphones with no music being played. IG (N = 38) - Underwent routine colonoscopy while listening to choice of music during procedure. | Choice of music from six genres (popular film songs, classical music, devotional, folk, instrumental, bioacoustics), *Headphones* | Pain, *VAS* Discomfort, *VAS* Willingness to repeat procedure, *Qualitative rating* | No significant between-group difference was identified in pain. Discomfort was significantly reduced in IG when compared to CG. Equal proportions of patients in both groups were willing to undergo repeat procedure using the same sedation protocol. |
| Hirani, *2024* | Colonoscopy, *Pakistan* | CG (N = 55) - Underwent routine colonoscopy. IG (N = 55) - In addition to CG care, received a music intervention from the time of randomization until transfer to recovery room. | Recordings of nature sounds combined with soft instrumental music, *Headphones* | Anxiety, *STAI* Pain, *VAS* | Pre-endoscopy anxiety scores were significantly reduced in IG (after a period of music therapy) when compared to CG. No significant between-group difference was identified in pain. |
| Hoya, *2007* | EGD, *Japan* | CG (N = 24) - Underwent routine EGD. IG (N = 26) - In addition to CG care, waited for 15 minutes prior to procedure in an "optimal soothing environment" comprising comforting images, sounds and smells. | Soothing recordings of nature sounds, *Speaker* | Anxiety, *VAS* | Anxiety was significantly increased from the time of arrival at the hospital to the time just before EGD in CG, whereas anxiety did not increase significantly over this same period in IG. No significant between-group difference was identified in causes of anxiety. |
| Kartin, *2017* | Colonoscopy, *Turkey* | CG (N = 30) - Underwent routine colonoscopy. IG (N = 30) - In addition to CG care, performed meditation for 10-15 minutes before colonoscopy and listened to a music of their choice during colonoscopy. | Choice of variety of music styles (e.g. classical, folk, pop), *Speaker* | Anxiety, *STAI*  Pain, *VAS and McGill pain questionnaire* | Anxiety decreased significantly from pre- to post-procedure in IG, whereas no significant change was identified in CG. There was a trend towards lower pain scores in IG when compared to CG, but the difference between the two groups was not significant. |
| Ko, *2017* | Colonoscopy, *Taiwan* | CG (N = 57) - Underwent routine colonoscopy without sedation. IG (N = 81) - In addition to CG care, received music therapy during colonoscopy. | Randomly assigned to either Canon in D by David Tolley (i.e. informal classical music) or Embracing the Wind by Kevin Kern (i.e. light music), *Speaker* | Anxiety, STAI | Anxiety scores were lower in IG when compared to CG, but the difference was not significant. No significant between-group differences were identified when controlling for sex. Anxiety scores were lowest amongst patients who listened to "light music", but again, the differences when compared to "informal classical music" and no music were not significant. |
| Ko, *2019* | Colonoscopy, *Hong Kong* | CG (N = 40) - Underwent routine colonoscopy. IG (N = 40) - In addition to CG care, listened to music for 20 minutes before and during the procedure. | Series of 15 "easy listening" non-rock Chinese songs, *Headphones* | Anxiety, *STAI* Pain, *VAS* Satisfaction, *VAS* | No significant between-group differences were identified in pain and anxiety. When compared with patients in CG, patients in IG reported higher levels of satisfaction with both the procedure and pain management. |
| Li, *2019* | Colonoscopy, *China* | CG (N = 48) - Underwent routine colonoscopy. IG-A (N = 46) - In addition to CG care, listened to "relaxing music" for 10 minutes before and during the procedure. IG-B (N = 50) - In addition to CG care, listened to a yoga nidra recording for 10 minutes before and during the procedure. | Mozart's Concerto Nos. 19, 21, 23 and 24 (i.e. relaxing music) or yoga nidra recording focused on breathing, holding and relaxing, *Headphones* | Pain, *NRS* Satisfaction, *NRS* Willingness to repeat procedure, *NRS* | Pain was significantly reduced in both IG-A and IG-B when compared to CG, with no significant difference identified between IG-A and IG-B. Satisfaction was significantly higher in both IG-A and IG-B when compared to CG, with the highest satisfaction identified in IG-B. Willingness to repeat the procedure was significantly greater in IG-B when compared to CG and IG-A. |
| Liu, *2024* | Colonoscopy, *China* | CG (N = 50) - Underwent routine colonoscopy. IG (N = 50) - In addition to CG care, received music therapy for one hour before and during the procedure. | Playlist of calming, soft melodies at a frequency of 200-500 Hertz, *Unknown* | Anxiety, *Self-rating anxiety scale* Pain, *VAS* | Anxiety was significantly reduced in IG when compared to CG. Pain was reduced in IG when compared to CG; however, this difference was not statistically significant. |
| Martindale, *2014* | Colonoscopy, *Australia* | CG (N = 17) - Underwent routine colonoscopy while wearing muted headphones. IG (N = 17) - Underwent routine colonoscopy while listening to music for 10 minutes prior to and during colonoscopy. | Music by Johann Sebastian Bach, *Headphones* | Anxiety, *STAI* Pain, *VAS* Satisfaction, *VAS* Willingness to repeat procedure, *VAS* Desire to have music therapy with repeat procedure, *Qualitative rating* | No significant between-group differences were identified in anxiety, pain, satisfaction or willingness to repeat the procedure. All patients in IG indicated that they would want music therapy again if they were to have a repeat procedure. |
| Meeuse, *2010* | Sigmoidoscopy, *Netherlands* | CG (N = 154) - Underwent routine sigmoidoscopy without sedation. IG (N = 153) - In addition to CG care, listened to music during the procedure. | Choice of various genres (e.g. classical, English pop, Dutch pop, jazz), *Headphones* | Pain, *VAS* | No significant between-group difference was identified in pain. A post hoc analysis failed to identify any demographic subgroups with a significant reduction in pain by listening to music. |
| Ovayolu, *2006* | Colonoscopy, *Turkey* | CG (N = 30) - Underwent routine colonoscopy. IG (N = 30) - In addition to CG care, listened to music before and during the procedure for a total of approximately 30 minutes. | Classical Turkish music involving a reed flute, *Speaker* | Anxiety, *STAI* Pain, *VAS* Satisfaction, *VAS* Comfort, *VAS* Willingness to repeat procedure, *VAS* | Anxiety and pain were significantly reduced in IG when compared to CG. Patients in IG also had significantly higher scores for satisfaction, comfort and willingness to repeat the procedure. |
| Padam, *2017* | EGD, *India* | CG (N = 66) - Underwent routine EGD, with a period of waiting without music for 10 minutes prior to the procedure. IG-A (N = 67) - Underwent routine EGD, with a period of listening to pre-recorded Vedic chants for 10 minutes prior to the procedure. IG-B (N = 66) - Underwent routine EGD, with a period of listening to Indian classical music for 10 minutes prior to the procedure. | Purusha Suktam (i.e. vedic chants) or Raga Kaushik Dwani Gat in Teentaal by Pandit Shivkumar (i.e. Indian classical music), *Headphones* | Anxiety, *STAI* | Anxiety levels were reduced in all groups from pre- to post-intervention, though the differences were found to be statistically significant in IG-A and IG-B. |
| Sobana, *2015* | EGD, *India* | CG (N = 30) - Underwent routine EGD, with a period of waiting without music for 10 minutes prior to the procedure. IG (N = 30) - Underwent routine EGD, with a period of listening to music for 10 minutes prior to the procedure. | Choice of songs from a catalogue of various genres (e.g. film music, devotional, relaxation), *Headphones* | Anxiety, *STAI* | Per the manuscript text, anxiety was significantly reduced from pre- to post-intervention in IG, whereas no significant pre- to post-intervention change was demonstrated in CG. That said, in reviewing the figures in the manuscript, it appears that anxiety scores actually increased in IG; thus, it is difficult to draw a clear conclusion from the results. |
| Spagnuolo, *2020* | EGD and/or colonoscopy, *Italy* | All groups underwent routine EGD and/or colonoscopy, with differences outlined below. CG-A (N = 121) - Conscious sedation without music. CG-B (N = 27) - Deep sedation without music. IG-A (N = 126) - Conscious sedation with music. IG-B (N = 37) - Deep sedation with music. | Music preferred by patients from three lists of songs grouped by genre (e.g. pop, classical, rock), *Headphones* | Anxiety, *STAI* Pain, *NRS* Satisfaction, *LS* Willingness to repeat endoscopy, *LS* | Anxiety and pain were significantly reduced in both intervention groups (IG-A and IG-B) when compared to their respective control groups (CG-A and CG-B). Scores for satisfaction and willingness to repeat the procedure were statistically greater in the intervention groups when compared to their respective control groups. |
| Sun, *2022* | Colonoscopy, *China* | CG (N = 104) - Underwent routine colonoscopy while wearing headphones without music playing. IG (N = 112) - Underwent routine colonoscopy while listening to piano music during the procedure. | Random light piano music, *Headphones* | Anxiety, *STAI* Pain, *VAS* Satisfaction, *VAS* Willingness to repeat endoscopy, *Qualitative rating* | Post-procedural state anxiety scores were significantly lower in IG when compared to CG. No significant between-group difference was identified in trait anxiety scores. When compared to CG, patients in IG reported significantly lower pain and significantly higher satisfaction. There was no significant between-group difference in willingness to repeat the procedure. |
| Sun, *2023* | Colonoscopy, *China* | CG (N = 76) - Underwent routine colonoscopy, with a period of waiting without music for 20 minutes prior to the procedure. IG-A (N = 76) - Underwent routine colonoscopy, with a period of listening to classical music for 20 minutes prior to the procedure. IG-B (N = 72) - Underwent routine colonoscopy, with a period of listening to pop music for 20 minutes prior to the procedure. | Selection of one-to-three classical songs (i.e. classical music) or popular songs (i.e. pop music) played on repeat, *Unknown* | Satisfaction, *VAS* Discomfort, *Qualitative rating* | Post-procedural satisfaction was significantly higher in IG-A and IG-B when compared to CG. No significant between-group difference was identified in reported discomfort. |
| Tani, *2022* | Colonoscopy, *Italy* | CG (N = 48) - Underwent routine colonoscopy. IG (N = 42) - In addition to CG care, listened to binaural beats for five minutes before and during the procedure. | Binaural beats with a binaural tone of 4 Hertz on a background of white noise, *Headphones* | Anxiety, *VAS* Pain, *VAS* Satisfaction, *LS* Willingness to repeat procedure, *LS* | No significant between-group differences were identified in anxiety. Pain was significantly reduced in IG when compared to CG. Scores for satisfaction and willingness to repeat the procedure were significantly higher in IG when compared to CG. |
| Walter, *2020* | Colonoscopy, *Germany* | CG (N = 98) - Underwent routine colonoscopy. IG (N = 98) - In addition to CG care, listened to self-chosen music during the procedure. | Patient-selected music using Spotify, *Headphones* | Satisfaction with sedation, *Patient satisfaction with sedation instrument* Satisfaction with music therapy, *Eight-item questionnaire with LS* | No significant between-group difference was identified in satisfaction with sedation. The authors proposed the ceiling effect as a potential cause of this finding, as the mean scores were greater than 90% in both groups. Qualitatively, patients in IG found music therapy to be pleasant and relaxing. |
| Yang, *2022* | EGD, *Taiwan* | CG (N = 100) - Underwent routine colonoscopy, with a period of waiting without music for 15 minutes prior to the procedure. IG (N = 100) - Underwent routine colonoscopy, with a period of listening to self-selected music for 15 minutes prior to the procedure. | Patient-selected music from various genres (e.g. Chinese pop, Taiwanese, western classical, light music, Western pop, nostalgic), *Headphones* | Anxiety, *STAI* | After adjusting for covariates, anxiety was significantly reduced in IG when compared to CG. In a post hoc analysis, the decrease in anxiety was found to be more pronounced amongst the subgroup in IG that self-identified to "strongly believe" in the beneficial effect of music on anxiety. |
